# Comparative Transcriptome Analyses Revealed Conserved and Novel Responses to Cold and Freezing Stress in *Brassica napus* L

**DOI:** 10.1534/g3.119.400229

**Published:** 2019-06-05

**Authors:** He Xin, Ni Xianchao, Xie Pan, Liu Wei, Yao Min, Kang Yu, Qin Lunwen, Hua Wei

**Affiliations:** * Southern Regional Collaborative Innovation Center for Grain and Oil Crops in China, Hunan Agricultural University, Changsha, Hunan, China 410128 and; † Key Laboratory of Biology and Genetic Improvement of Oil Crops, Ministry of Agriculture, Oil Crops Research Institute of the Chinese Academy of Agricultural Sciences, Wuhan, China

**Keywords:** Transcriptome, rapeseed, cold acclimation, cold shock, chilling, freezing

## Abstract

Oil rapeseed (*Brassica napus* L.) is a typical winter biennial plant, with high cold tolerance during vegetative stage. In recent years, more and more early-maturing rapeseed varieties were planted across China. Unfortunately, the early-maturing rapeseed varieties with low cold tolerance have higher risk of freeze injury in cold winter and spring. Little is known about the molecular mechanisms for coping with different low-temperature stress conditions in rapeseed. In this study, we investigated 47,328 differentially expressed genes (DEGs) of two early-maturing rapeseed varieties with different cold tolerance treated with cold shock at chilling (4°) and freezing (−4°) temperatures, as well as chilling and freezing stress following cold acclimation or control conditions. Kyoto Encyclopedia of Genes and Genomes (KEGG) analysis indicated that two conserved (the primary metabolism and plant hormone signal transduction) and two novel (plant-pathogen interaction pathway and circadian rhythms pathway) signaling pathways were significantly enriched with differentially-expressed transcripts. Our results provided a foundation for understanding the low-temperature stress response mechanisms of rapeseed. We also propose new ideas and candidate genes for genetic improvement of rapeseed tolerance to cold stresses.

Low-temperature is a major environmental stress that adversely affects plant growth and development, limiting crop productivity. Plants evolutionarily developed a wide variety of mechanisms to cope with low-temperature stress. The response to low-temperature in plants is a complex process that involves morphological, physiological, biochemical and molecular processes, including gene expression, enzyme activity and metabolic homeostasis (Theocharis *et al.* 2012; Nievola *et al.* 2017). Chilling stress (0-15°) causes the membrane to rigidify, destabilizes protein complexes and impairs photosynthesis, whereas freezing stress (< 0°) causes more serious injuries to the plant (Shi *et al.* 2018; Ruelland *et al.* 2009).

Many plants showed increased freezing tolerance upon exposure to low non-freezing temperatures in a phenomenon known as cold acclimation (Thomashow 1999). Cold acclimation involved an array of physiological and biochemical modifications, and these altered processes involved changes in gene expression patterns via phytohormone and the ICE-CBF-COR transcriptional regulatory cascade (Shi *et al.* 2018).

C-repeat binding factors (CBF) transcription factors, also known as dehydration responsive element binding factor 1 (DREB1) proteins, could bind directly to the CRT/DRE *cis*-element in the promoters of cold regulated (*COR*) genes, and subsequently induced the expression of *COR* genes (Jaglo-Ottosen *et al.* 1998; Liu *et al.* 1998). The *COR* genes protected plant cells against cold-induced damage, repaired cold-rigidified membranes and stabilized cellular osmotic potential by encoding cryoprotective proteins and key enzymes for osmolyte biosynthesis (Chinnusamy *et al.* 2007). In *Arabidopsis*, the basic helix-loop-helix (bHLH) transcription factor ICE1 was shown to bind directly to the CANNTG *cis*-elements in the promoters of CBF and upregulated CBF expression under cold stress (Chinnusamy *et al.* 2003; Kim *et al.* 2015). Apart from CBFs, various transcriptional factors including HSFC1, ZAT12, ZF, ZAT10 and RAV1 were also involved in modulating COR expression by bypassing CBF signaling (Park *et al.* 2015).

Abscisic acid (ABA) is a vital plant hormone that plays a key role in stress resistance during plant growth and development (Vishwakarma *et al.* 2017). It was reported that OST1/SnRK2E, a serine-threonine protein kinase in ABA core signaling pathway, acted upstream of CBFs to positively regulate freezing tolerance via phosphorylating ICE1 to prevent its 26S proteasome-mediated degradation by HOS1 (Ding *et al.* 2015). SnRK2E phosphorylated basic transcription factors 3 (BTF3) and BTF3-like factors, and facilitated their interactions with CBFs to promote CBF stability under cold stress (Ding *et al.* 2018).

Jasmonic acid (JA) is a lipid-derived plant hormone that plays an important role in the plant abiotic and biotic stresses (Wasternack and Song 2017; Wasternack and Strnad 2018). It was reported that JA signaling positively regulated the plant responses to freezing stress via the interaction between the repressors of jasmonate signaling proteins (TIFY/JAZ) and ICE1/2, thus suppressing the transcriptional activity of ICE proteins, thereby attenuating CBF genes expression (Hu *et al.* 2013).

Oil rapeseed is a winter biennial oil-seed crop, responds to vernalization and shows an excellent tolerance to cold stresses during vegetative stage. In recent years, more and more early-maturing rapeseed varieties were planted across China. Unfortunately, the early-maturing rapeseed varieties with low cold tolerance have higher risk of freeze injury in cold winter and spring. Hence, it is vital to compare early-maturing rapeseed varieties tolerant to cold and evaluate molecular mechanisms that adapt to different low-temperature stress conditions.

In this study, RNA-seq technology was used to compare differentially expressed genes (DEGs) of two early-maturing rapeseed varieties with different cold tolerance, that were treated with cold shock (4° and −4°) temperatures, as well as chilling (4°) and freezing (−4°) temperatures following cold acclimation and control conditions. Analyses identified conserved and novel signaling pathways and genes. Our results provided a foundation for understanding the low-temperature stress response mechanisms of rapeseed. We also propose new ideas and candidate genes for genetic improvement of rapeseed tolerance to cold stresses.

## Materials and Methods

### Plant materials and treatments

The seedlings of two early-maturing semi-winter rapeseed varieties (HX17 and HX58) were cultured in incubators under 20° (14 h light: am6:00-pm8:00)/16° (10 h dark: pm8:00-am6:00) 4 weeks, then treated with 4° (14 days) →4° (12 h) or - 4° (12 h), 20°/16° (light/dark) 6 weeks → 4° (12 h), 20° (14 h light: am6:00-pm8:00)/16° (10 h dark: pm8:00-am6:00) 6 weeks →- 4° (12 h). For the acclimation condition, after the 14 days at 4°, 4°/-4° (12h) mean a treatment with 4° or -4° at pm8:00-am8:00 (10 h dark and 2 h light). Then the third leaves from the top were collected at am8:00 after cold treatment and stored at - 80° immediately until RNA extraction.

### Total RNA extraction and sequencing

The samples were sent to the sequencing cooperation of Novogene Co. Ltd (Bejing, China) for RNA isolation, examination and sequencing. HiSeq PE150 of Illumina was selected for the sample transcriptome sequencing.

### Transcriptome assembly and annotation

Raw data (raw reads) of fastq format were first processed through in-house perl scripts. In this step, clean data/reads were obtained by removing reads containing adapter or, reads containing ploy-N and low quality reads from raw data. At the same time, Q20, Q30 and GC content for the clean data were calculated. All the downstream analyses were based on the clean data with high quality.

Reference genome and gene model annotation files were downloaded from genome website directly. Index of the reference genome was built using Hisat2 v2.0.4 and paired end reads were aligned to the reference genome using Hisat2 v2.0.4. Hisat2 was selected as the mapping tool because it can generate a database of splice junctions based on the gene model annotation file, thus providing better mapping result than other non-splice mapping tools.

HTSeq v0.9.1 was used to count the reads mapped to each gene. FPKM of each gene was calculated based on the length of the gene and read counts mapped to the gene. FPKM considers the combined effects of sequencing depth and gene length for the read counts, and is currently the most commonly used method for estimating gene expression levels (Trapnell *et al.* 2009).

### Differential expression analyses of transcripts

Differential expression analysis of two conditions/groups (three biological replicates per condition) was performed using the DESeq R package (1.18.0). DESeq provide statistical routines for determining differential expression in digital gene expression data using a model based on the negative binomial distribution (K_ij_ ∼ NB(μ_ij_,σ_ij_^2^)). The resulting *P*-values were adjusted using the Multiple Hypothesis Testing for controlling false discovery rate. Genes with an adjusted *P*-value < 0.05 calculated by DESeq were assigned as differentially expressed.

### GO and KEGG enrichment analyses of differentially expressed genes (DEGs)

Gene ontology (GO) enrichment analysis of DEGs was implemented by the GOseq R package, in which gene length bias was corrected. GO terms with corrected *P*-value < 0.05 were considered significantly enriched in DEGs.

KEGG is a database resource for understanding high-level functions and utilities of the biological system, such as the cell, the organism and the ecosystem, at molecular level, especially large-scale datasets generated by genome sequencing and other high throughput technologies (http://www.genome.jp/kegg/). KOBAS software was used to test the statistical significance of enrichment of DEGs in KEGG pathways.

### qRT-PCR

To confirm the validity of the RNA-Sequencing data, we randomly selected nine DEGs for qRT-PCR analysis. 3 µg total RNAs/sample was used for cDNA biosynthesis with Superscript III reverse transcriptase (Invitrogen, San Diego, USA). The qRT-PCR was performed using the CFX96 Real-Time System (Bio-Rad, USA) with SYBR green (Bio-Rad, USA). The relative changes were calculated with 2-∆Ct and the *BnaActin* (*BnaC05g34300D*) from *B. napus* was used as the endogenous reference gene. The relative transcript level was determined and normalized using the reference level and averaged over the three technical replicates. Primers for the qRT-PCR (Table S10) were designed according to the cDNAs sequences using Primer Premier 5 (http://www.premierbiosoft.com/crm/jsp/com/pbi/crm/clientside/ProductList.jsp).

### Data Availability

The sequencing rawdata are accessible through GEO (GSE129220): https://www.ncbi.nlm.nih.gov/geo/query/acc.cgi?acc=GSE129220. Supplemental material available at Figshare: https://10.6084/m9.figshare.7379858.

## Results and discussion

### Transcriptomic analyses

Two early-maturing semi-winter rapeseed varieties (HX17 and HX58) with different cold tolerance (Figure S1) were selected for transcriptomic investigation by RNA-Seq following different low-temperature treatments.

**Figure 1:**
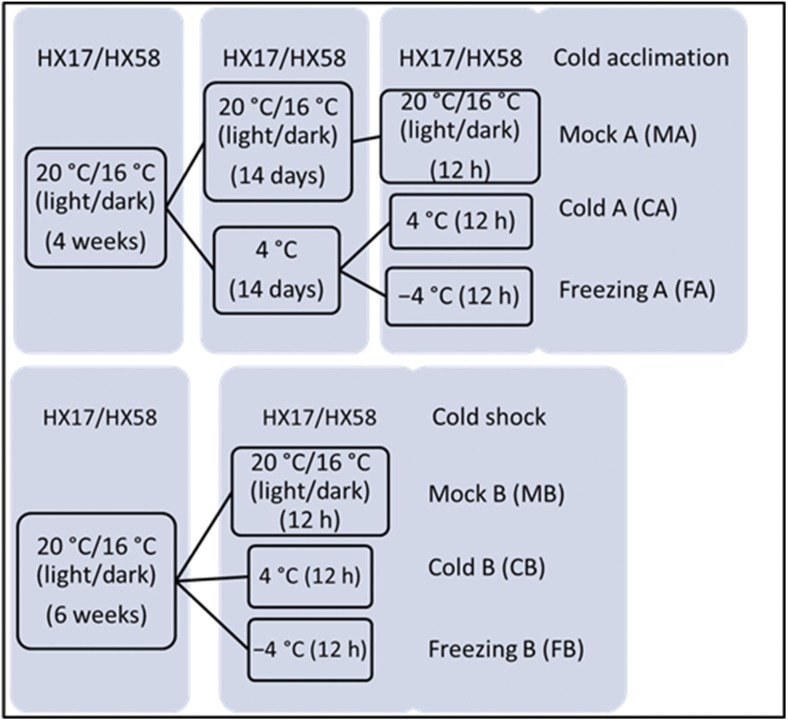
The processes of different low-temperature treatments on two rapeseed (*Brassica. napus L*. cv. HX17 and HX58) seedlings.

The responsive mechanisms were different between chilling and freezing temperatures, as well as cold shock and cold acclimation in plants (Ruelland *et al.* 2009; Guy 1999; Nievola *et al.* 2017). A short-term exposure to low, non-freezing temperatures sufficiently induced cold acclimation resulting in increased tolerance to freezing conditions [5]. Accordingly, 20°/16° (light/dark) →cold-acclimation (4°, 14 days) → 4°/−4° (12 h), 20°/16° (light/dark) → 4°/−4° (12 h), were selected as the treatment conditions (Figure 1).

Total RNA was extracted from the seedlings of HX17 and HX58 treated with different low-temperature conditions and sequenced using the Illumina HiSeq 2500 platform. A total of 296.67 Gb clean data (1,977,933,478 clean reads) were acquired after removal of adaptor sequences, ambiguous nucleotides and low-quality sequences (Table 1). Approximately 43.1-78.1 million clean reads were obtained from the libraries. The Q-score for 88.17–93.99% of reads was Q30, and 82.57–86.51% of the total reads were uniquely mapped to the reference genome (Table S1). The mapped sequences were assembled with Cufflinks, referenced against the annotated genome sequence of the tetraploid species *B. napus* provided by Index of /brassicanapus/data (http://www.genoscope.cns.fr/brassicanapus/data/). RNA-Seq revealed that there was a total of 172,412 transcripts and 110,367 unigenes, and 10,578 genes on average for each sample, including 78,945 known genes and 26,233 novel genes on average (Table S2). Gene expression levels were estimated by fragments per kilo base of transcript per million fragments mapped (FPKM) (Langmead *et al.* 2009). Approximately 8.51–11.97% of total genes’ FPKM was more than 15.0, and 59.26–65.50% of total genes’ FPKM was lower than 1.0 (Table S3).

**Table 1:** Quality filtering and statistics of raw reads obtained in transcriptome libraries of B. napus exposed to low-temperature treatments

Sample name	Raw reads	Clean reads	clean bases	Error rate (%)	Q20 (%)	Q30 (%)	GC content (%)
HX17_MA1	61608094	58966572	8.84G	0.03	97.36	92.93	44.06
HX17_MA2	46842038	45252272	6.79G	0.03	96.98	92.17	44.39
HX17_MA3	51620482	50405900	7.56G	0.03	97.11	92.01	45.91
HX17_CA1	45028428	44096512	6.61G	0.03	96.97	91.66	45.83
HX17_CA2	53540226	52633174	7.89G	0.03	96.72	91.49	46.35
HX17_CA3	49776538	47962390	7.19G	0.03	96.70	91.54	43.39
HX17_FA1	46114476	45390992	6.81G	0.03	96.66	91.37	44.84
HX17_FA2	66471914	64442766	9.67G	0.03	97.11	92.40	45.85
HX17_FA3	49756338	49191330	7.38G	0.03	97.20	92.53	45.64
HX17_MB1	45873106	45028940	6.75G	0.03	95.42	88.17	44.09
HX17_MB2	47889494	47067140	7.06G	0.03	96.75	91.57	45.00
HX17_MB3	46228026	45344394	6.80G	0.03	96.94	92.04	44.25
HX17_CB1	70149774	68467860	10.27G	0.03	97.85	94.12	44.40
HX17_CB2	54975576	53977612	8.10G	0.03	97.12	92.04	44.50
HX17_CB3	55136028	53840712	8.08G	0.03	97.02	91.82	44.74
HX17_FB1	79518680	78163306	11.72G	0.03	97.70	93.70	44.76
HX17_FB2	48126142	47147508	7.07G	0.03	96.75	91.28	44.80
HX17_FB3	52485614	51527762	7.73G	0.03	97.77	93.79	44.19
HX58_MA1	67391320	65727088	9.86G	0.03	97.16	92.16	44.28
HX58_MA2	66677164	64776152	9.72G	0.03	97.09	91.98	43.86
HX58_MA3	47017928	44892330	6.73G	0.03	97.07	92.07	43.88
HX58_CA1	44491830	43128444	6.47G	0.03	96.66	91.39	46.46
HX58_CA2	47877742	46243718	6.94G	0.03	96.73	91.40	45.62
HX58_CA3	49056666	47594184	7.14G	0.03	97.85	93.99	45.77
HX58_FA1	77825480	76080646	11.41G	0.03	97.92	94.08	44.84
HX58_FA2	72714882	70675296	10.6G	0.03	97.72	93.61	45.33
HX58_FA3	62524530	60787534	9.12G	0.03	97.71	93.59	45.10
HX58_MB1	55247676	54098192	8.11G	0.03	96.74	91.23	44.28
HX58_MB2	57837196	56764648	8.51G	0.03	96.82	91.34	45.16
HX58_MB3	49353542	48225898	7.23G	0.03	97.34	92.54	44.69
HX58_CB1	61754716	60061840	9.01G	0.03	97.88	93.95	43.42
HX58_CB2	66709668	64672348	9.70G	0.03	97.69	93.60	42.76
HX58_CB3	52648416	51400508	7.71G	0.03	96.73	91.28	43.77
HX58_FB1	58213716	56978060	8.55G	0.03	97.04	91.89	46.07
HX58_FB2	59190884	57729982	8.66G	0.03	97.11	92.37	46.39
HX58_FB3	60769766	59189468	8.88G	0.03	97.10	92.36	45.06

Assembly Steps	Total assembled transcripts	Number of transcripts (>1000bp)	Percent of transcripts	Maximum transcript length	Average transcript length	N50 value (bp)	Coverage (X)
Assembler SOAPdenovotrans (65 k-mer)	91,765	32,509	35.43	12,805	903.39	1,427	160
After gap filling and hierarchical clustering	53,806	24,429	45.40	12,674	1,096.96	1,499	218
After removing mis-assembled transcripts	46,556	19,347	41.55	9,811	1,013.66	1,393	226
BLASTX best group representatives (unigenes)	25,400	12,588	49.56	9,811	1,153.30	1,502	240

For the evaluation of DEGs’ reliability and the filter of abnormal samples, Pearson correlation coefficient (PCC) analysis was conducted. The correlation analysis indicated that all three technical replicates replication samples showed more than 0.929 (R^2^) of similarities (Table S4).

### Differentially expressed genes (DEGs) in rapeseed under different low-temperature treatments

Differential expression analysis of treatments and control group was performed using the DESeq (Anders and Huber 2010). A threshold of fold change of ≥2 and p-adjusted q-value of < 0.005 was used for identifying DEGs.

**Figure 2:**
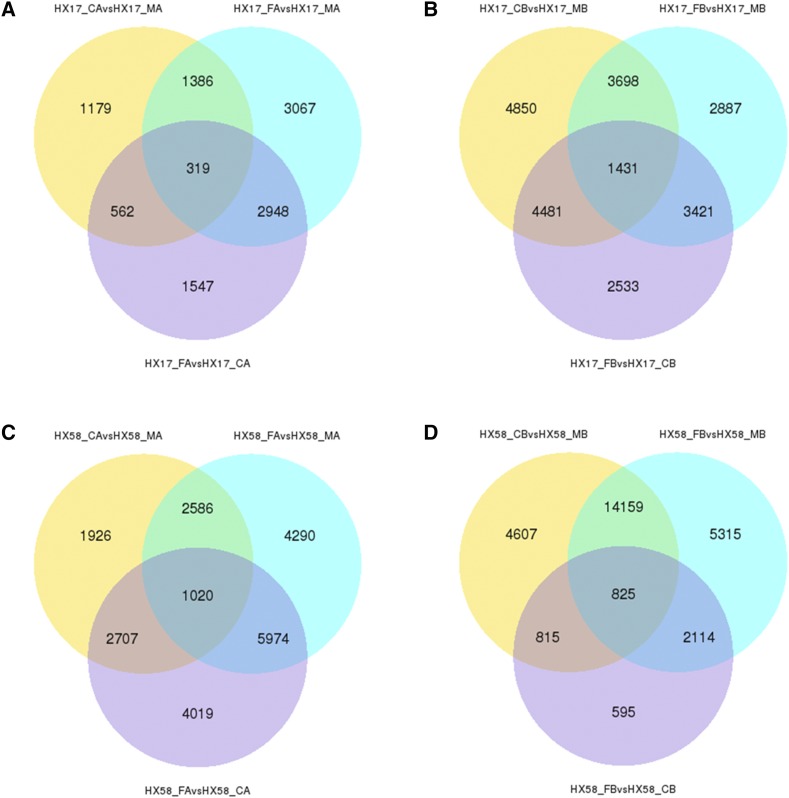
Venn diagram of DEGs between HX17_MA, HX17_CA, HX17_FA (A); HX17_MB, HX17_CB, HX17_FB (B); HX58_MA, HX58_CA, HX58_FA (C); HX58_MA, HX58_CA, HX58_FB (D).

**Figure 3:**
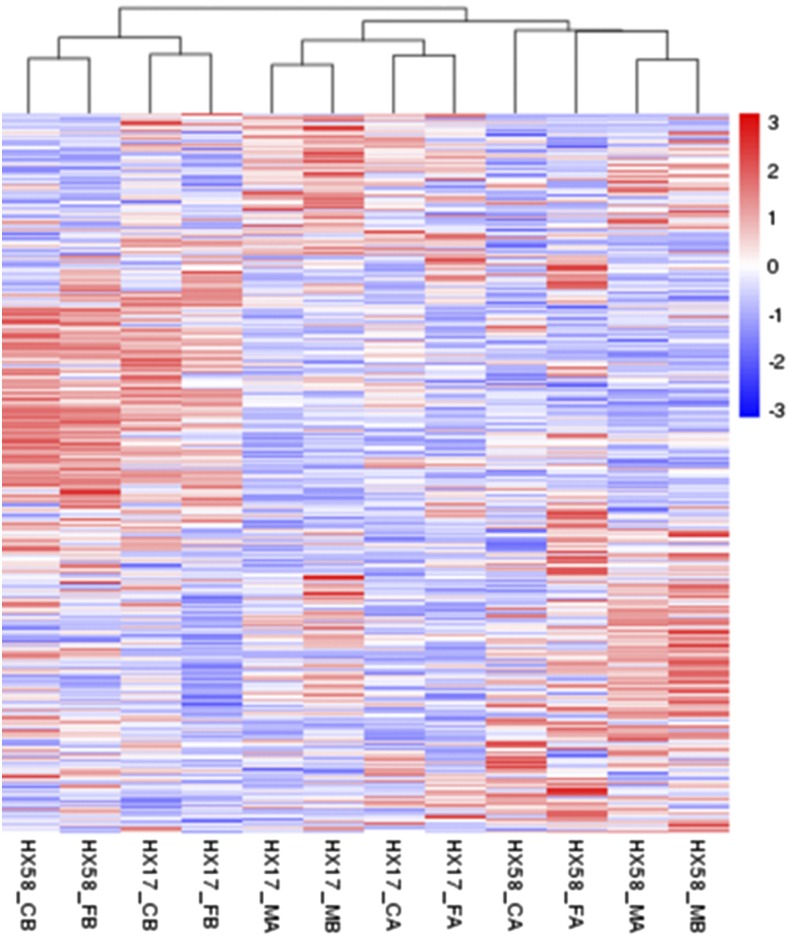
Hierarchical cluster analysis of differentially expressed genes (DEGs) among all the 12 samples. Heatmap of DEGs among the 12 samples. Red indicates high expression, and blue indicates low expression. Color from red to blue represents descending log10 (FPKM + 1).

A total of 47,328 DEGs (HX17_CA *vs.* HX17_MA: 3446; HX17_FA *vs.* HX17_MA: 7738; HX17_FA *vs.* HX17_CA: 5378; HX17_CB *vs.* HX17_MB: 16802; HX17_FB *vs.* HX17_MB: 22271; HX17_FB *vs.* HX17_CB: 3704; HX58_CA *vs.* HX58_MA: 10340; HX58_FA *vs.* HX58_MA: 16505; HX58_FA *vs.* HX58_CA: 18280; HX58_CB *vs.* HX58_MB: 21902; HX58_FB *vs.* HX58_MB: 25279; HX58_FB *vs.* HX58_CB: 4381) were identified in the different low-temperature treatments (Figure 2 and Table S5). Statistically, DEGs in both HX17 and HX58 showed that FB had the most DEGs and CA had the least, in the order of FB > CB > FA> CA. HX58 presented with more DEGs than HX17 (Figure 2), consistent with the cluster analysis of DEGs (Figure 3 and Figure S2). In addition, the lower temperature correlated with more injuries, and HX58 was more susceptible to low-temperature stress (Figure S1). DEGs under different stresses in both HX17 and HX58 were presented as CA *vs.* FA > CB *vs.* FB (Figure 2), confirming that the cold acclimation altered the responses to freezing stress in plant.

### Pathway analyses of responses to different low-temperature stresses in rapeseed

Pathway analyses of DEGs against the Kyoto Encyclopedia of Genes and Genomes (KEGG) contributed to the understanding of gene functions (Kanehisa *et al.* 2008). As shown in Figure S3 and Table S6, there was no enriched pathway in both HX17 and HX58 treated with cold shock (CB and FB) compared to control condition (MB), indicating that cold shock stress caused serious injuries to the plant. Interestingly, there was no enriched pathway in HX58 acclimatized to cold (CA and FA), similar to cold shock stress. However, the plant hormone signal transduction pathway was enriched in HX17 acclimatized to cold (CA and FA), indicating that the plant hormone signal transduction was activated by cold acclimation in cold-tolerant rapeseed. Further analyses of the upregulated and downregulated DEGs enriched pathways are reported in Table S7. Pathways involved in primary metabolism and plant hormone signal transduction were enriched under different low-temperature stresses in both HX17 and HX58 (Fig. S3, Table S6). Previous studies also reported changes in these two pathways in various crops and plants exposed to low-temperature stresses (Nievola *et al.* 2017; Qu *et al.* 2015; Zhang *et al.* 2017; Li *et al.* 2017; Bai *et al.* 2015; Zhang *et al.* 2014), suggested that low-temperature stresses were conservative to affect the normal metabolism of plants and the plant hormone signal transduction.

**Figure 4:**
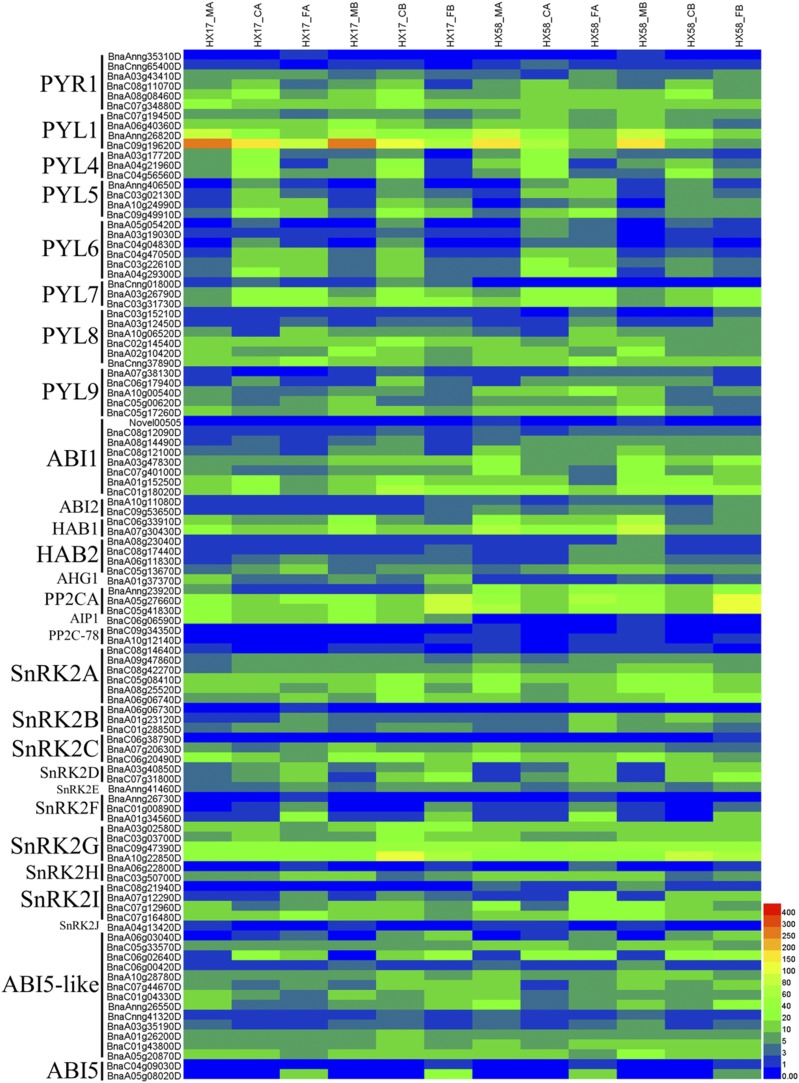
Heatmap analysis of the genes in ABA signal pathway. PYR1 (PYRABACTIN RESISTANCE 1), Abscisic acid receptor; PYL (PYR1-like protein), Abscisic acid receptor; ABI1/2 (ABSCISIC ACID-INSENSITIVE 1/2), Protein phosphatase 2C; HAB1/2 (HYPERSENSITIVE TO ABA 1/2), Protein phosphatase 2C; AHG1 (ABA-HYPERSENSITIVE GERMINATION 1), Protein phosphatase 2C; PP2C, Protein phosphatase 2C; AIP1 (AKT1-INTERACTING 1), Protein phosphatase 2C; SnRK2 (SNF1-related kinase 2), Serine/threonine-protein kinase; ABI5 (ABSCISIC ACID-INSENSITIVE 5), bZIP transcription factor. The expression levels of each gene (FPKM values) in each sample, as indicated by different color rectangles. Red means high expression; Blue means low expression.

There were many reports that the plant hormone signal pathways played important roles in the plant responses to abiotic stresses (Colebrook *et al.* 2014; Kazan 2015; Gururani *et al.* 2015; Bielach *et al.* 2017; Verma *et al.* 2016; de Zelicourt *et al.* 2016; Khan *et al.* 2015; Sharma *et al.* 2017). The ABA and JA biosynthesis and signaling were induced by various abiotic stresses, and positively regulated the low-temperature tolerance in plants (Yoshida *et al.* 2014; Huang *et al.* 2017; Hu *et al.* 2017; Shinkawa *et al.* 2013; Hu *et al.* 2013; Du *et al.* 2013; Wang *et al.* 2016a). Hence, we analyzed the expression patterns of ABA and JA signaling genes. The results showed that among the 503 DEGs of the plant hormone signaling transduction (total of 1148 genes), 94 of them were the ABA signaling genes (total of 139 genes) and 43 of them were JA signaling genes (total of 61 genes)(Table S7). Most of the ABA signaling genes were changed under cold acclimation and cold shock treatments in both HX17 and HX58 but they presented different expression profiles (Figure 4). The ABA receptors pyrabactin resistance proteins/PYR-like proteins/regulatory components of ABA receptors (PYR/PYL/RCAR) *PYL5* and *PYL7* were both induced by cold and freezing treatments in HX17 and HX58, while the *PYL1* and *PYL9* were suppressed in all treatments. The *PYR1* and *PYL4* were only induced by cold treatment but not by freezing treatment. The *PYL6* were induced by cold treatment and freezing treatment following cold acclimation. ABA co-receptor phosphatase 2C (PP2C) *ABI1* and *HAB1* were suppressed by all treatments, while *HAB2* was upregulated. The Snf1-related protein kinase 2 (SnRK2) *SnRK2B* and *SnRK2D* were induced by all low-temperature treatments, while *SnRK2C* was suppressed. Interestingly, *SnRK2F* was induced only by freezing treatment, similar to *ABI5* expression, indicating that they played important roles in response to freezing in rapeseed. The *Arabidopsis* ABI5 was a bZIP transcription factor phosphorylated by SnRK2D and SnRK2I, and participated in ABA-regulated gene expression during seed development and subsequent vegetative stage by acting as the major mediator of ABA repression of growth (Skubacz *et al.* 2016; Nakashima *et al.* 2009; Brocard *et al.* 2002). It is possible that SnRK2F directly phosphorylated ABI5 in rapeseed. The *SnRK2E/OST1* was induced only in HX17 but not in HX58, which may be a part reason for the better cold-tolerance of HX17 than HX58.

**Figure 5:**
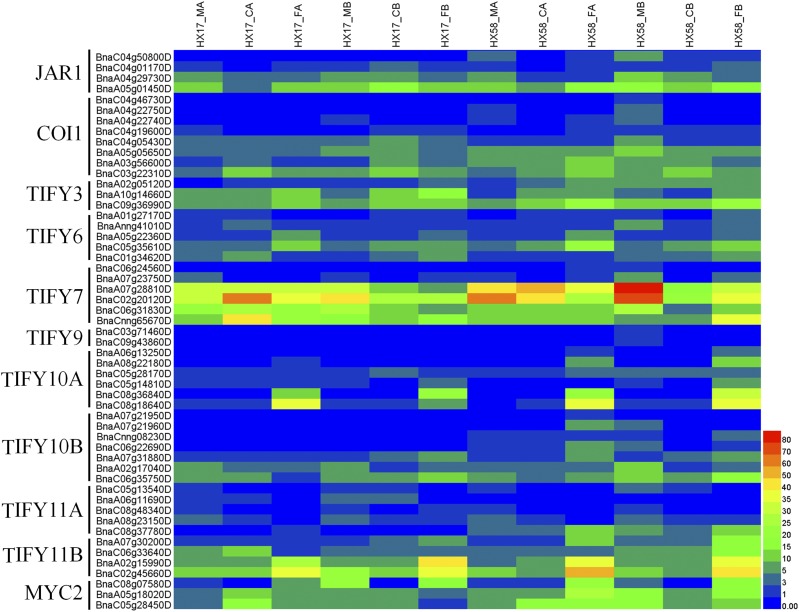
Heatmap analysis of the genes in JA signal pathway. JAR1 (JASMONATE RESISTANT 1), Jasmonic acid-amido synthetase; COI1 (Coronatine-insensitive protein 1), Jasmonic acid receptor; TIFY, Jasmonate ZIM domain-containing protein; MYC2, bHLH transcription factor MYC2. The expression levels of each gene (FPKM values) in each sample, as indicated by different color rectangles. Red means high expression; Blue means low expression.

It was reported that exogenous application of JA enhances plant freezing tolerance with or without cold acclimation, and overexpression of *JAZ1/TIFY10A* and *JAZ4/TIFY6* suppressed the transcriptional function of ICE1, thereby attenuating the ICE-CBF-COR signaling pathway and freezing responses in *Arabidopsis* (Hu *et al.* 2013). As shown in Figure 5, the expressions of many JA signaling genes were altered in both HX17 and HX58 under low-temperature conditions. *TIFY3* and *TIFY6* were induced in both HX17 and HX58 under all low-temperature conditions. Interestingly, *TIFY7* was suppressed only under cold shock treatments, while the *TIFY10A* and *TIFY11B* were upregulated only by freezing treatments, similar to *SnRK2F* and *ABI5* (Figure 4 and Figure 5). These changes suggest that they may be important regulators in the freezing response in rapeseed. Additionally, *MYC2* was only induced by cold acclimation, but not by cold shock.

**Figure 6:**
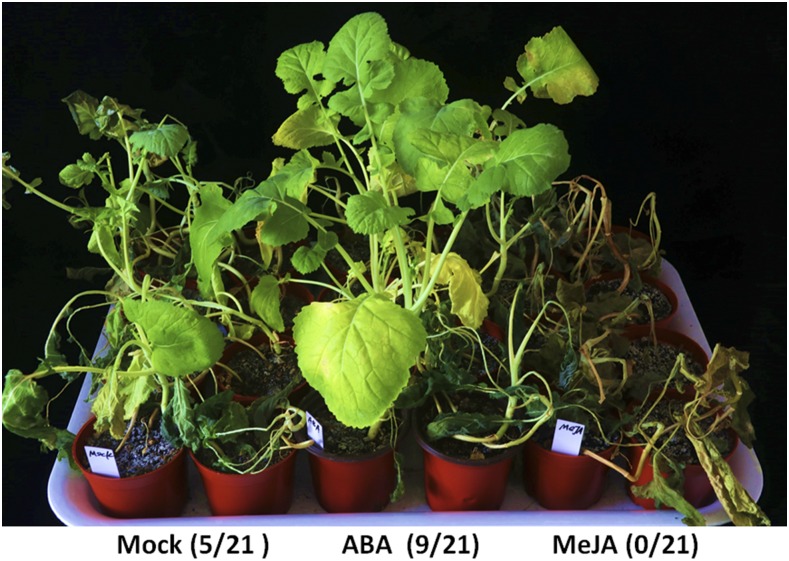
Effect of ABA and MeJA on the freezing tolerance of ZS11 (Brassica. napus L. cv. Zhongshuang 11) seedlings. ZS11 seedlings were grown for 4-week under normal conditions at 20 °C, and then sprayed with ABA (100 μM), MeJA (100 μM) or water (Mock) for 1 day. Then the seedlings were treated with -6 °C for 2 days and recovered under normal conditions at 20 °C for 7 days. Survival rates of seedlings were shown in brackets.

To test whether exogenous ABA and methyl-jasmonic acid (MeJA) could improve the rapeseed cold tolerance, 4-week-old rapeseed seedlings were sprayed with ABA (100 μM), MeJA (100 μM) and water before cold shock at -6°. The survival rate results demonstrated that exogenous ABA significantly improved the rapeseed freezing tolerance. On the contrary, exogenous MeJA reduced the rapeseed freezing tolerance (Figure 6). The concentration of exogenous MeJA might be too high, affecting the rapeseed freezing tolerance. Other studies reported that treatment with exogenous JA induced leaf senescence and expression of senescence associated genes, while exogenous high concentration MeJA caused plant injures (Zhu *et al.* 2015). All the results indicated that a complex gene regulatory network composed of plant hormones signaling affected the low-temperature stresses in rapeseed, and appropriate concentrations of exogenous plant hormones could increase rapeseed’s cold tolerance.

### Identification of the transcriptional regulatory network responding to low-temperature stress in rapeseed

Transcription factors (TF) play a central role in the gene regulatory networks that mediate various aspects of plant developmental processes and responses to environmental changes (Hoang *et al.* 2017; Riechmann and Ratcliffe 2000). The program iTAK was used to analyze DEGs and identified 7633 TFs. Of these, 3670 (48.1%) belong to 76 transcription factors and transcriptional regulator families. The 10 top families were MYB, AP2-EREBP, bHLH, Orphans, HB, bZIP, NAC, C2H2, WRKY and C3H (Table S8).

**Figure 7:**
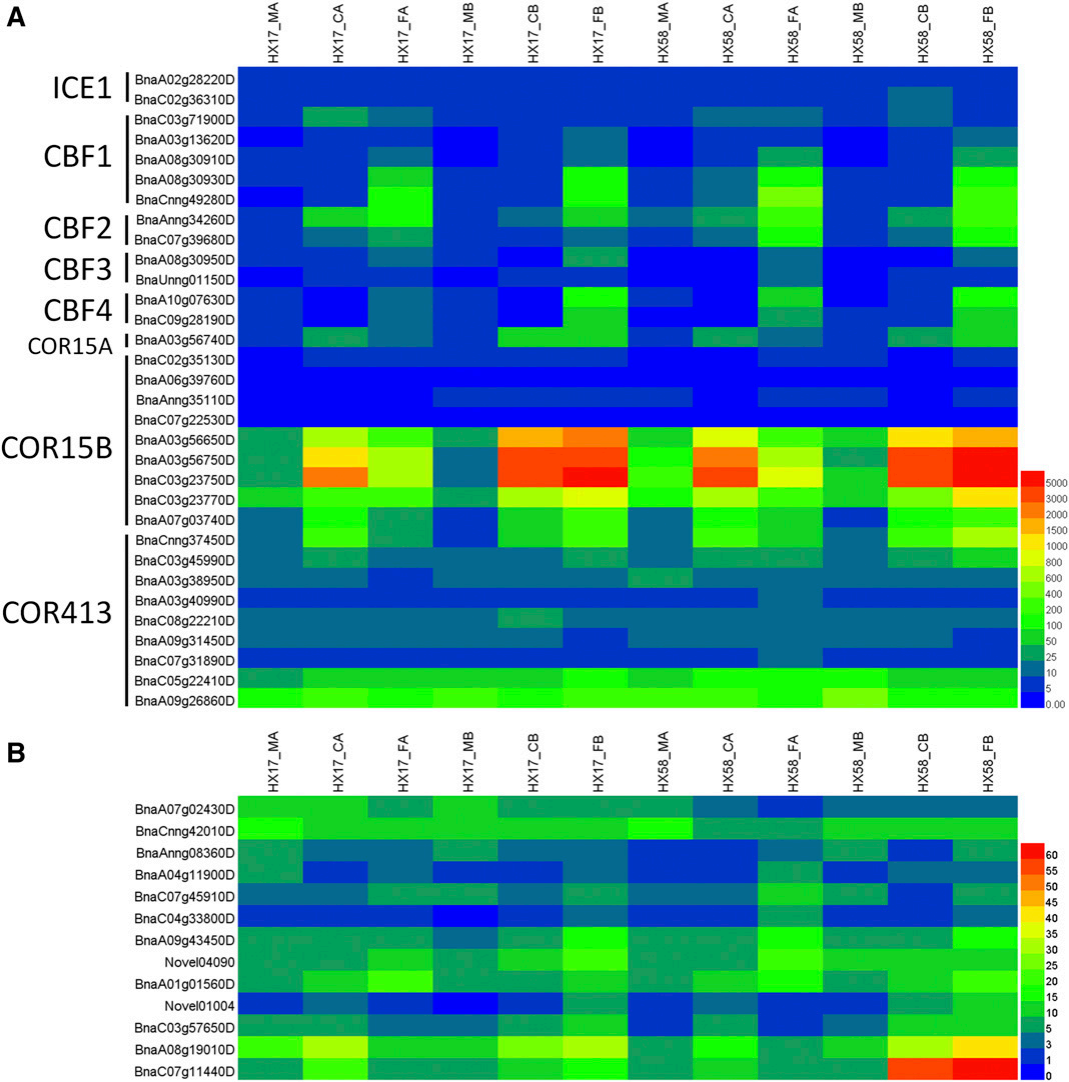
Heatmap analysis of the ICE-CBF-COR signal pathway (A) and cold shock domain-containing proteins (B). ICE1 (Inducer of CBF expression 1), bHLH transcription factor; CBF (C-repeat-binding factor), dehydration-responsive element-binding protein; COR, cold-regulated. The expression levels of each gene (FPKM values) in each sample, as indicated by different color rectangles. Red means high expression; Blue means low expression.

It was reported that CBFs were the core regulator in the cold-response signaling in plants (Shi *et al.* 2018; Guo *et al.* 2018; Ruelland *et al.* 2009; Wang *et al.* 2017). CBFs belong to the DREB1 proteins, including DRE1A/CBF3, DRE1B/CBF1, DRE1C/CBF2 and DRE1D/CBF4 (Liu *et al.* 1998; Jaglo-Ottosen *et al.* 1998; Sakuma *et al.* 2002). We analyzed the *ICE1*, *CBFs* and some *COR* genes, and observed that *COR15A* and *COR15B* were significantly induced by all low-temperature treatments (Figure 7A). Apart from BnaC03g71900D, all the *CBF* genes were induced by freezing stress more than chilling stress, regardless of cold acclimation (Figure 7A), highlighting the importance of *CBFs* in rapeseed freezing tolerance. However, *ICE1* was not induced by low-temperature treatments, as opposed to *AtICE1* (Chinnusamy *et al.* 2003).

The cold shock domain-containing proteins (CSP) act as RNA chaperones that destabilize mRNA secondary structures at low temperatures, and they are regulated by low-temperature stress (Sasaki and Imai 2011; Choi *et al.* 2015; Radkova *et al.* 2014; Chaikam and Karlson 2008). Overexpression of *AtCSP2* resulted in decreased freezing tolerance despite cold acclimation (Sasaki *et al.* 2013). CSP functions are associated mainly with cold adaptation, but they are also involved in other biological processes under normal growth conditions (Sasaki and Imai 2011; Nakaminami *et al.* 2009; Park *et al.* 2009). All the 13 cold *CSPs* were regulated by the low-temperature treatments (Figure 7B). As shown in Figure 7B, three *CSP* genes (*BnaA07g02430D*, *BnaCnng42010D*, and *BnaAnng08360D*) were suppressed by low-temperature treatments, and the others were induced by at least one treatment. *BnaA01g01560D* was induced by all low-temperature treatments. Four *CSP* (*Novel01004*, *BnaC03g57650D*, *BnaA08g19010D*, and *BnaC07g11440D*) were induced by cold shock (CB and FB) and chilling after cold acclimation (CA), but not freezing after acclimation (FA). *BnaA09g43450D* and *Novel04090* were induced by cold shock (CB and FB) and chilling after cold acclimation (FA), but not freezing after acclimation (CA). All the results indicated that the *CSP* genes have a complex expression pattern in response to different low-temperature conditions.

Heat stress transcription factors (HSFs) play a crucial role in plant responses to high-temperature by regulating the expression of stress-responsive genes, such as heat shock proteins (Guo *et al.* 2016). Approximately half of HSFs were affected under low-temperature condition (Table S8), suggesting that HSFs responded to high-temperature and low-temperature changes.

### Plant-pathogen interaction pathways play important roles in low-temperature stresses in rapeseed

**Figure 8:**
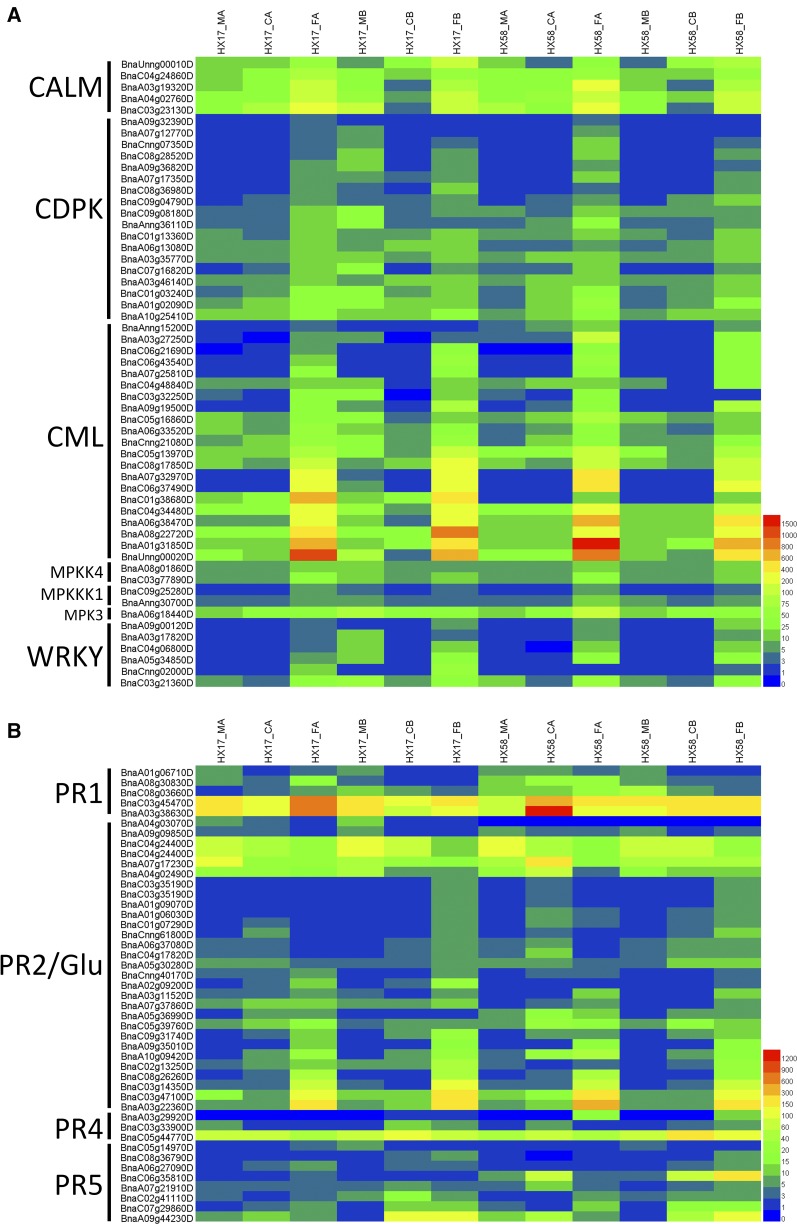
Heatmap analysis of the regulator genes (A) and PR genes (B) in the plant-pathogen interaction pathway. CAML, Calmodulin, CDPK; Calcium-dependent protein kinase; CML, calcium-binding protein; MPK3, Mitogen-activated protein kinase 3; MPKK4, Mitogen-activated protein kinase kinase 4; MPKKK1, Mitogen-activated protein kinase kinase kinase 1; WRKY, WRKY transcription factor; PR, Pathogenesis-related protein; Glu, Glucan endo-1,3-beta-glucosidase. The expression levels of each gene (FPKM values) in each sample, as indicated by different color rectangles. Red means high expression; Blue means low expression.

As shown in Table S6-S7, the plant-pathogen interaction pathway was enriched in both HX17 and HX58 under low-temperature conditions, especially under the freezing stresses, consistent with expression of the plant-pathogen interaction pathway genes (Figure 8). Even though the plant-pathogen interaction pathway was consistently reported to be enriched under low-temperature treatments in various plants (Zhang *et al.* 2017; Qu *et al.* 2015; Yang and Huang 2018; Xu *et al.* 2016; Wang *et al.* 2016b; Tian *et al.* 2013; Du *et al.* 2015; Wang *et al.* 2015), the functions of this pathway in the plant cold responsiveness were neglected by researchers. The pathogenesis-related proteins (PR) were induced by cold acclimation (Kuwabara and Imai 2009; Hincha *et al.* 1997). Pre-treatment of tomato fruit with MeJA or MeSA induced the synthesis of PR proteins, which led to increased chilling tolerance and resistance to pathogens (Ding *et al.* 2002). Overexpression of 3 PR genes (*PR2*, *PR4*, *Glu*) in *Arabidopsis* enabled the plant to tolerate freezing temperatures (Cabello *et al.* 2012). As shown in Figure 8b, *PR1* and *PR5* were induced by cold acclimation and cold shock. Overexpression of a rice *PR5* gene in rapeseed enhanced the resistance to *Sclerotinia sclerotiorum*, a fungal pathogen that caused Sclerotinia stem rot in rapeseed (Aghazadeh *et al.* 2017). We propose that overexpression of *PR* genes in rapeseed might produce rapeseed germplasm with tolerance to cold and *S. sclerotiorum*.

### Circadian rhythms play an important role in low-temperature stresses in rapeseed

**Figure 9:**
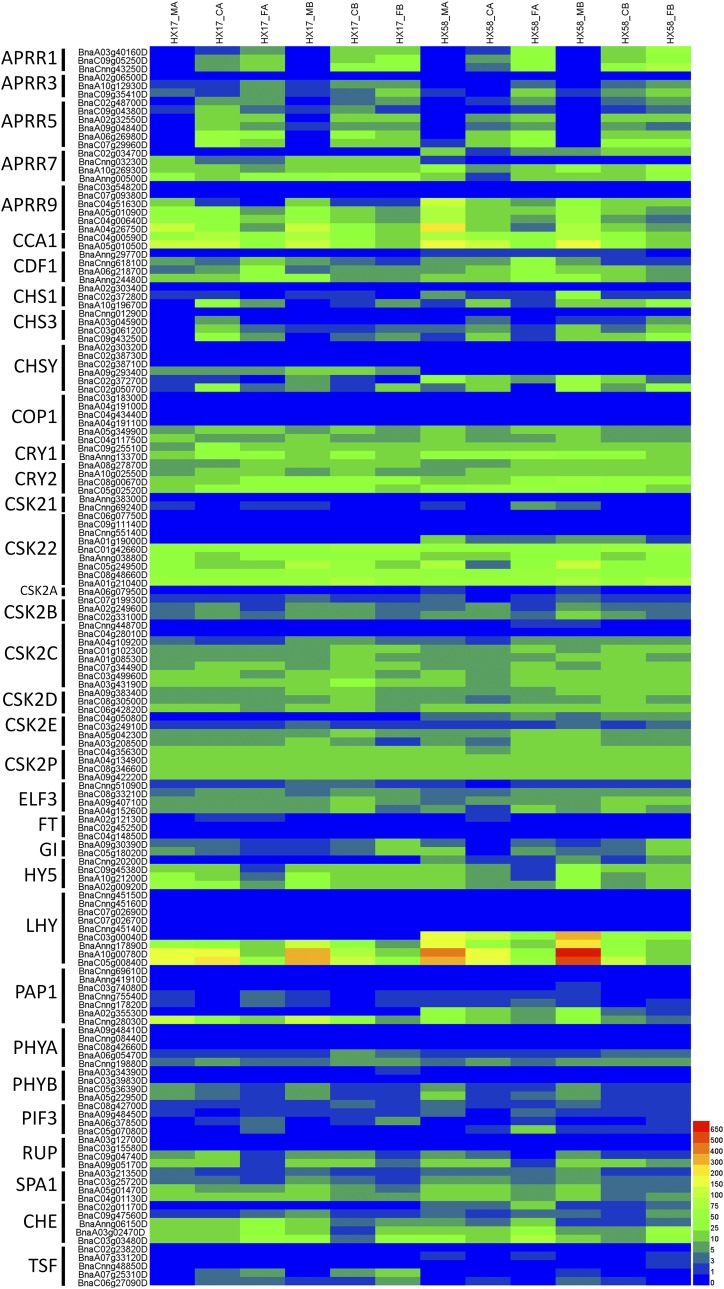
Heatmap analysis of the genes in circadian rhythms pathway. APRR (pseudo response regulator), Two-component response regulator-like; CCA1 (CIRCADIAN CLOCK ASSOCIATED 1), MYB-related transcription factor; CDF1 (Cyclic dof factor 1), Dof zinc finger protein; CHS (Chalcone synthase); COP1 (Constitutive photomorphogenesis protein 1), E3 ubiquitin-protein ligase; CRY1/2 (Cryptochrome 1/2), Blue light photoreceptor; CSK2, Casein kinase II subunit; ELF3 (EARLY FLOWERING 3); FT (FLOWERING LOCUS T), Phosphatidylethanolamine-binding protein; GI (GIGANTEA); HY5 (ELONGATED HYPOCOTYL5), bZIP transcription factor; LHY (LATE ELONGATED HYPOCOTYL), MYB-related transcription factor; PAP1(Production of anthocyanin pigment 1 protein), MYB-related transcription factor; PHYA/B (Phytochrome A/B), ; PIF3 (Phytochrome-interacting factor 3), bHLH transcription factor; RUP1, WD repeat-containing protein; SPA1 (SUPPRESSOR OF PHYA-105 1), WD repeat-containing protein; CHE (CCA1 HIKING EXPEDITION), TCP transcription factor; TSF (TWIN SISTER of FT). The expression levels of each gene (FPKM values) in each sample, as indicated by different color rectangles. Red means high expression; Blue means low expression.

As shown in Table S6-S7, the circadian rhythms pathway was enriched in the DEGs for both HX17 and HX58 under low-temperature conditions. It was also shown to respond to low-temperature conditions in several plants (Li *et al.* 2017; Sobkowiak *et al.* 2014; Liu *et al.* 2018; Abeynayake *et al.* 2015; Barah *et al.* 2013), and regulated CBFs and CORs gene expression (Edwards *et al.* 2006; Bieniawska *et al.* 2008). Almost all the genes associated with circadian rhythms showed differential expression (Figure 9). These included induction of the pseudo-response regulators (APRR) *APRR1/TOC1*, *APRR3* and *APRR5* by chilling and freezing stresses in both HX17 and HX58, and suppression of *APRR7* and *APRR9*, consistent with the reports on *Arabidopsis* (Lee *et al.* 2005). A prr7-3 prr9-1 double mutant failed to entrain to temperature cycles (Salome and McClung 2005), while prr5/7/9 triple mutant plant was more tolerant to cold stresses through upregulation of CBF expression (Nakamichi *et al.* 2009). The Cholcone synthase (*CHS*), twin sister of FT (*TSF*), phytochrome A (*PHYA*) and transcription factor *PIF3* were induced by chilling and freezing stresses. The circadian clock associated 1 (*CCA1*), early flowering 3 (*ELF3*), late elongated hypocotyl (*LHY*), phytochrome B (*PHYB*), suppressor of PHYA-105 1 (*SPA1*), repressor of UV-B photomorphogenesis 1 (*RUP*) and transcription factors *HY5* and *MYB75* were suppressed by chilling and freezing stresses. Cyclic dof factor 1 (*CDF1*) and cytochrome 1/2 (*CRY1/2*) were induced by cold acclimation, but were suppressed by cold shock. Gigantea (*GI*) and *CHE/TCP21* were induced only by freezing stress.

Two phytochrome mutants phyB and phyD showed increased cold tolerance via the upregulation of CBF regulon (Franklin and Whitelam 2007). During the warm long-day condition growing season, the CBF pathway is actively suppressed by PHYB, PIF4, and PIF7 (Lee and Thomashow 2012). PHYA and PHYB function antagonistically to regulate cold tolerance via abscisic acid-dependent jasmonate signaling in tomato (Wang *et al.* 2016a). PIF3 acts as a negative regulator of plant cold acclimation by direct suppression of CBF expression (Jiang *et al.* 2017).

HY5 transcription was regulated by low temperature through a CBF- and ABA-independent pathway, and mutant hy5-1 provoked a significant reduction in the ability of *Arabidopsis* to cold acclimate (Catala *et al.* 2011). GI increased freezing tolerance via a CBF-independent pathway (Cao *et al.* 2005). CCA1/LHY bound to promoter of CBFs and promoted cold acclimation (Dong *et al.* 2011). COR27 and COR28 were direct targets of CCA1, meanwhile they bind to the chromatin of APRR1 and APRR5 and repress their expression (Li *et al.* 2016). Therefore, we could infer that the circadian rhythm pathways play a role in freezing tolerance, through regulation of the circadian rhythm genes in rapeseed.

### Vernalization in rapeseed

Although both cold acclimation and vernalization are responses to low temperature, the durations of cold exposure required to initiate these responses are quite distinct. The most upstream event of the vernalization pathway identified is the induction of vernalization insensitive 3 (VIN3) in response to low temperatures. Together with VIL1, VIN3 was required during vernalization for the modifications of FLC and FLM chromatin, it led to an epigenetically silenced state and acquisition of competence to flower (Sung and Amasino 2004).

**Figure 10:**
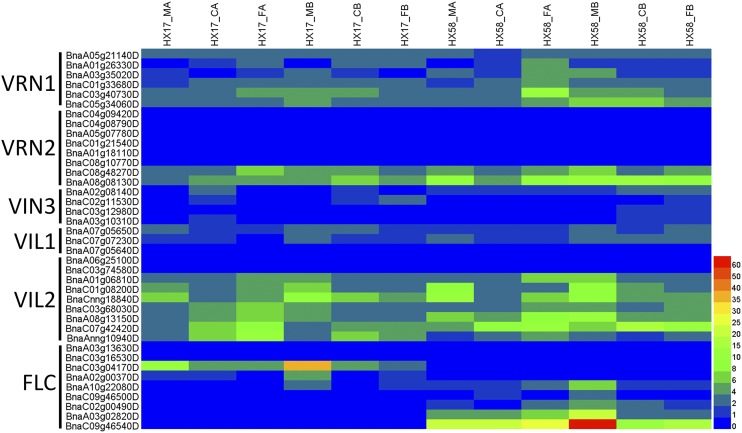
Heatmap analysis of the genes in the vernalization pathway. VRN1 (VERNALIZATION 1), B3 domain-containing transcription factor; VRN2 (VERNALIZATION 2), Zinc finger protein; VIN3 (VERNALIZATION INSENSITIVE 3), PHD finger protein; VIL, VIN3-like protein; FLC (FLOWERING LOCUS C), MADS-box protein. The expression levels of each gene (FPKM values) in each sample, as indicated by different color rectangles. Red means high expression; Blue means low expression.

We analyzed the expression pattern of all rapeseed *VIN* and *FLC* genes under different low-temperature conditions. As shown in Figure 10, only two *VIN* genes (*BnaC07g42420D* and *BnaAnng10940D*) were induced by cold acclimation and cold shock. *BnaC01g08200D* and *BnaCnng18840D* were suppressed by both cold acclimation and cold shock. *BnaC03g68030D* and *BnaA08g13150D* were induced only by cold acclimation but not by cold shock. The induction of a few *VIN* genes in response to low temperatures suggested that the vernalization pathway in early-maturing rapeseed was attenuated.

The expression patterns of nine rapeseed *FLCs* were different between HX17 and HX58 (Figure 10). Two *FLC* genes were suppressed only in HX17 under cold-acclimation and cold-shock, while three *FLC* genes were suppressed only in HX58. *BnaA10g22080D* was suppressed in both HX17 and HX58 under cold shock stresses. All the results suggest that *FLC* genes vary extensively among the rapeseed populations.

### Validation of gene expression patterns by qRT-PCR

**Figure 11:**
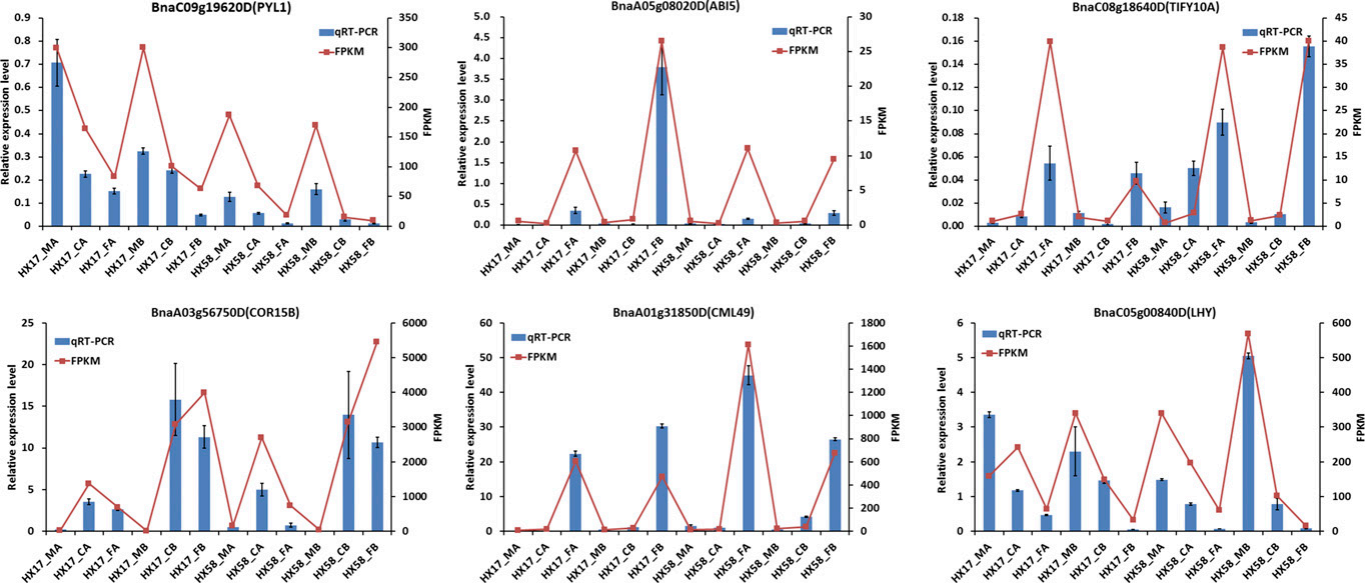
qRT-PCR verification of 6 selected DEGs. Comparison of qRT-PCR (blue bar) with FPKM data (red line). The relative changes were calculated with 2-∆Ct and the rapeseed *BnaActin* gene was amplified as the reference gene. The relative qRT-PCR expression level (selected DEG/ *BnaActin*) is shown on the left y-axis. The relative transcript level was determined and normalized using the reference level and averaged over the three technical replicates. The FPKM from the RNA-Seq data are indicated on the right y-axis.

As shown in Figure 11, all the 4 candidate genes were induced by freezing stress strongly, with or without cold acclimation. Although the fold-changes in their expression detected by sequencing did not exactly match those detected by qRT-PCR, the detected expression patterns were mostly consistent for all the selected genes, confirming the reliability of the RNA-Seq results.

### The differences in cold tolerance between HX58 and HX17

**Figure 12:**
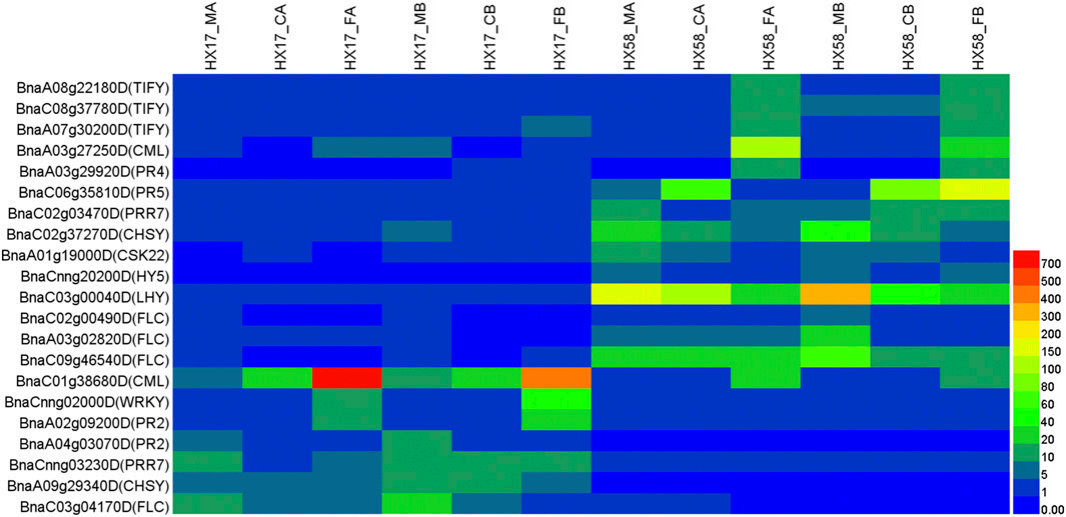
Heatmap analysis of the DEG genes between HX58 and HX17. The expression levels of each gene (FPKM values) in each sample, as indicated by different color rectangles. Red means high expression; Blue means low expression.

As shown in Figure 12, there are some genes involved in JA signaling, Ca^2+^-signaling, plant-pathogen interaction pathway, circadian rhythms pathway and flowering pathway with different expression patterns and levels between HX58 and HX17, and this may suggest that those genes’ expression extent and amount lead the difference in cold tolerance between HX58 and HX17.

## Conclusions

This study is the first report of the transcriptome data of two early-maturing rapeseeds under different low-temperature stresses (cold acclimation and cold shock; chilling and freezing) using HiSeq PE150 of Illumina. A total of 47,328 DEGs were identified in rapeseed treated with different low temperatures. Further analysis of these DEGs showed that the low-temperature response was a complex process in rapeseed. Many KEGG pathways were enriched following low-temperature treatments, including the primary metabolism, plant hormone signal transduction, plant-pathogen interaction pathway, circadian rhythms and so on. These results provide a reference for our understanding of the rapeseed adaptation to different low-temperature environments and provide new insights into the molecular mechanisms of different low-temperature tolerance in rapeseed.
