# Erratum to: Comparative Transcriptome Analyses Revealed Conserved and Novel Responses to Cold and Freezing Stress in *Brassica napus* L

**DOI:** 10.1093/g3journal/jkab108

**Published:** 2021-04-19

**Authors:** Xin He, Xianchao Ni, Pan Xie, Wei Liu, Min Yao, Yu Kang, Lunwen Qin, Wei Hua

*G3: Genes|Genomes|Genetics*, Volume 9, 2723–2737, https://doi.org/10.1534/g3.119.400229

In the originally published version of this manuscript, the authors’ names were listed incorrectly. The correct author list is: Xin He, Xianchao Ni, Pan Xie, Wei Liu, Min Yao, Yu Kang, Lunwen Qin, Wei Hua. These details have been corrected only in this erratum to preserve the published version of record.

